# Prenatal Ultrasound Screening for Corpus Callosum Anomalies: A Narrative Review

**DOI:** 10.3390/diagnostics16172721

**Published:** 2026-08-26

**Authors:** Kwok-Yin Leung

**Affiliations:** Obstetrics and Gynaecology, Gleneagles Hospital Hong Kong, Hong Kong, China; ky@kyleung.org

**Keywords:** callosal dysgenesis, corpus callosum, obstetric ultrasound, screening, second trimester, sonography, three-dimensional

## Abstract

Prenatal detection of agenesis of the corpus callosum (CC) anomalies is a challenge. Although a correct diagnosis of anomalies of the CC requires direct examination of the CC in the mid-sagittal plane of the foetal brain, the international guidelines on the mid-trimester morphology scan do not recommend such direct examination of the CC in low-risk populations because of the associated technical difficulties. Recently, such routine direct assessment has been recommended by several international experts in a consensus statement. The implementation of such routine direct assessment is not easy given the difficulties encountered in obtaining the mid-sagittal view of the CC. As such, it is the time to revisit the various two-dimensional and three-dimensional ultrasound techniques with a view to improve visualisation of the CC. The aim of this narrative review article is to discuss the use of various prenatal ultrasound screening methods of CC anomalies including standard axial views, more detailed axial views, the mid-sagittal view, the transvaginal approach, and 3D reconstruction. New insights on screening methods, and a pragmatic approach are also shared.

## 1. Introduction

The corpus callosum (CC) is the largest commissure connecting the two cerebral hemispheres and consists of decussating interhemispheric axons that permit learning and memory to be shared between the cerebral hemispheres [1]. The CC consists of four components, namely, the rostrum, the genu, the corpus and the splenium [2].

It is feasible to visualise the CC on prenatal ultrasound in the mid-sagittal view of the foetal brain, as a narrow anechoic area, delineated superiorly and inferiorly by two echogenic lines, overlaying the quadrigeminal plate of the mesencephalon [3]. The CC forms the roof of three structures, namely, the frontal horns, the cavum septi pellucidi (CSP), and the bodies of the lateral ventricles [4]. The frontal horns, CSP, and third ventricle are imaged as anechoic spaces deep to the CC [5]. The pericallosal sulcus (and its vessels) is seen as a thin, hyperechoic line that separates the CC from the cingulate gyrus [5]. The latter appears as a broad hypoechoic band that is cocurvilinear with the CC and lies one level more superficial [5].

Prenatal detection of agenesis of CC (ACC) or other CC anomalies is important as they are among the most common malformations affecting the central nervous system (CNS) with a reported prevalence ranging from 3 to 7 per 1000 births [6]. Additionally, they have a strong association with chromosomal abnormalities, genetic syndromes, and neurodevelopmental delay [7]. Even isolated partial ACC is associated with mild-to-severe disabilities including speech disorders, behaviour and motor deficit disorders [8]. Anomalies of the CC can occur in isolation or be associated with other cerebral, structural, and genetic abnormalities [9,10]. A recent study showed that 43% of prenatally diagnosed ACC had a pathogenic/likely pathogenic variant identified on exome sequencing following a negative chromosomal microarray analysis [10].

The prognosis of ACC is highly variable and can range from normal outcomes to abnormal outcomes including severe intellectual disability, epilepsy and motor impairment [3,11,12]. The prognosis depends on whether it is isolated or associated with other structural or genetic abnormalities [12]. In general, foetuses with isolated ACC have a better prognosis than those with non-isolated ACC [11]. Still, the rate of abnormal neurodevelopmental outcomes in infants with a prenatal diagnosis of isolated ACC is about 25–30% [3]. There was no significant difference in the severe disability rate between isolated complete and partial ACC according to some small-scale studies [3,7,8]. Furthermore, around 15% of apparently ‘isolated’ cases prenatally were found to have associated abnormalities postnatally [3]. Unpredictable neurodevelopmental outcomes may occur in truly isolated ACC, and mild disabilities may develop in older children [11].

Prenatal diagnosis of the CC anomalies is a challenge and can cause anxiety to the parents [11,12,13]. First, although a correct diagnosis of anomalies of the CC requires direct examination of the CC in the mid-sagittal plane of the foetal brain, the International Society of Ultrasound in Obstetrics and Gynecology (ISUOG) guidelines on the mid-trimester morphology scan published in 2021, and on the screening examination of the foetal CNS in 2020 do not recommend such direct examination of the CC in low-risk populations because of the associated technical difficulties [14,15]. Instead, ISUOG recommends three standard axial views of the foetal brain, which can be easily obtained for screening examination, and can detect indirect signs of CC anomalies [14,15], but the comma-shaped CC is not well visualised in these axial views. However, a review of twelve studies involving 544 foetuses with suspected anomalies of CNS showed that the pooled sensitivity of prenatal ultrasound diagnosis of ACC was 0.72 only [16]. To improve the prenatal diagnosis of partial ACC, examination of the anterior and posterior complex has been proposed since 2015 [17]. Furthermore, a step-by-step approach has recently been recommended to get the most from the axial views of the foetal brain to improve the detection of indirect signs of CC anomalies, among other brain anomalies [18].

Second, it may be difficult to visualise the CC on prenatal two-dimensional (2D) ultrasound in the mid-sagittal view of the foetal brain because of unfavourable foetal head position, calcification of the cranium, foetal movements, and the thick maternal abdominal wall [19,20]. To overcome these difficulties, three-dimensional (3D) ultrasound reconstruction of the mid-sagittal plane has been proposed as an alternative approach for the evaluation of the CC [19,20,21].

Third, a review of foetal pathologies of the CC reported in the prenatal imaging literature over a period of almost 30 years showed large heterogeneity in the definitions and terminology used to describe similar pathologies [22]. To achieve a more standardised classification system with agreed criteria, Solomon et al. proposed a simple classification: complete ACC, partial ACC, or dysraphic CC [23]. The absence of all four segments of the CC is called complete ACC whereas the absence of one or more of the four segments is called partial ACC [23,24]. Solomon et al. also suggested to avoid using complicated ultrasound descriptions and simple sub-threshold biometry because they can generate considerable anxiety experienced by the parents-to-be of a foetus with a suspected or confirmed CC anomaly [23].

To improve the prenatal screening for CC anomalies, routine direct assessment of the CC with the mid-sagittal view has been recommended by several international experts in a recent consensus statement [25]. The implementation of such routine direct assessment is not easy given the difficulties encountered in obtaining the mid-sagittal view of the CC [19,20]. As such, it is the time to revisit the various 2D and 3D ultrasound techniques with a view to improve visualisation of the CC.

This is a narrative review. Databases including PubMed and Google were searched by using keywords including callosal dysgenesis; corpus callosum; obstetric ultrasound; screening; second trimester; sonography; and three-dimensional. Relevant articles, including guidelines, observational studies, and reviews published from 1 January 2006 to 1 April 2026, were included. Inclusion criteria included: (a) studies or meta-analyses on prenatal 2D or 3D ultrasound screening or diagnosis of complete or partial ACC, or other CC anomalies, and (b) international guidelines, consensus statements or review by experts on the prenatal detection of CC anomalies. Exclusion criteria included: case reports or series with fewer than three cases of CC anomalies. Publications including the ascertainment of CC anomalies and postnatal neurodevelopment were selected when prognosis was discussed.

The aim of this review article is to discuss the use of various prenatal ultrasound screening methods of CC anomalies including the standard axial views, more detailed axial views, the mid-sagittal view, the transvaginal approach, and 3D reconstruction. New insights on screening methods are also shared.

## 2. Standard Approach: Axial Views

**Figure 1:**
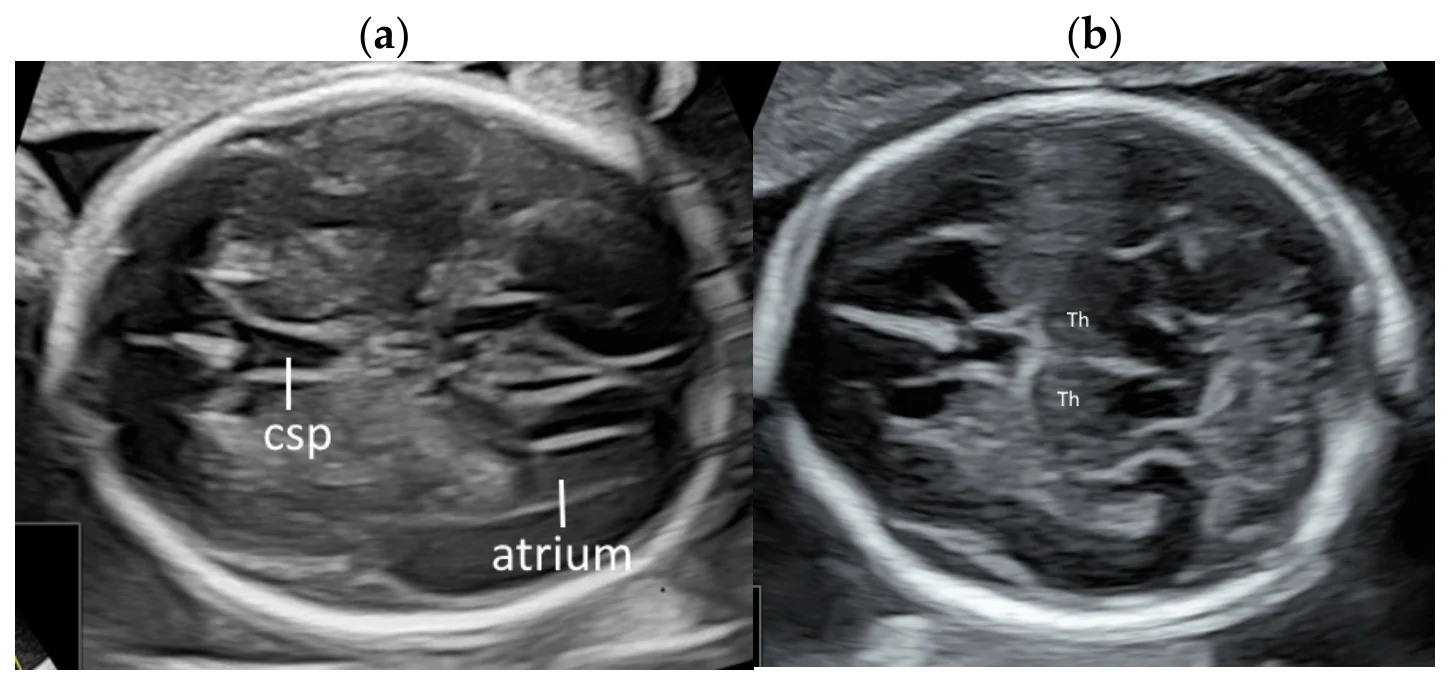
Transverse views of the foetal head, demonstrating the standard trans-ventricular (**a**), and trans-thalamic (**b**) scanning planes. These planes allow detection of indirect signs of agenesis of the corpus callosum. CSP, cavum septi pellucidi; Th, thalamus.

According the ISUOG Guidelines [14,15], the standard scanning planes for the basic examination of the foetal brain include three axial planes, namely, the trans-ventricular plane, the trans-thalamic plane, and the trans-cerebellar plane. The lateral ventricles, CSP, midline falx, and thalami should be evaluated [14] to look for “indirect” signs of CC anomalies (Figure 1a,b).

These indirect signs include an abnormal or absent CSP, dilatation, or a tear-shaped appearance and malalignment of the lateral ventricles, a high-riding and dilated third ventricle (between right and left thalami), and a widened interhemispheric fissure [24,26,27]. If any of these signs is found, a targeted foetal neurosonographic examination should be performed [15].

However, there are limitations of using these indirect signs to detect ACC. In a study of 118 cases of partial ACC, prenatal ultrasound revealed the presence of an abnormal CSP, distention of the interhemispheric fissure, a dilated and elevated third ventricle, and ventriculomegaly in 86.4%, 77.1%, 47.4%, and 35.6% of cases, respectively, in the transverse plane of the brain [28].

Furthermore, these indirect signs can be subtle and sometimes absent at all before 24 weeks of gestation, particularly in the diagnosis of partial ACC [24,29,30]. A square-shaped CSP or tear-shaped ventricles may not be obvious to an inexperienced sonographer [31]. Additionally, a high-riding and dilated third ventricle can be mistaken as the CSP [24]. Furthermore, a third of ultrasound examinations in foetuses with partial ACC may not show any abnormality in trans-ventricular screening view before 24 weeks [24]. In particular, no ventriculomegaly or absence of the CSP was found [24]. Thus, the diagnosis of ACC may be missed at the mid-trimester screening ultrasound [24]. Although cases of complete ACC or a large partial ACC are more easily detected by the standard axial views of the foetal brain; cases with a small partial ACC may easily be missed [32]. In my opinion, the standard approach is insufficient to prenatally detect ACC, especially partial ACC.

## 3. A More Detailed Axial Views than the Standard Approach

### 3.1. Anterior and Posterior Complex

**Figure 2:**
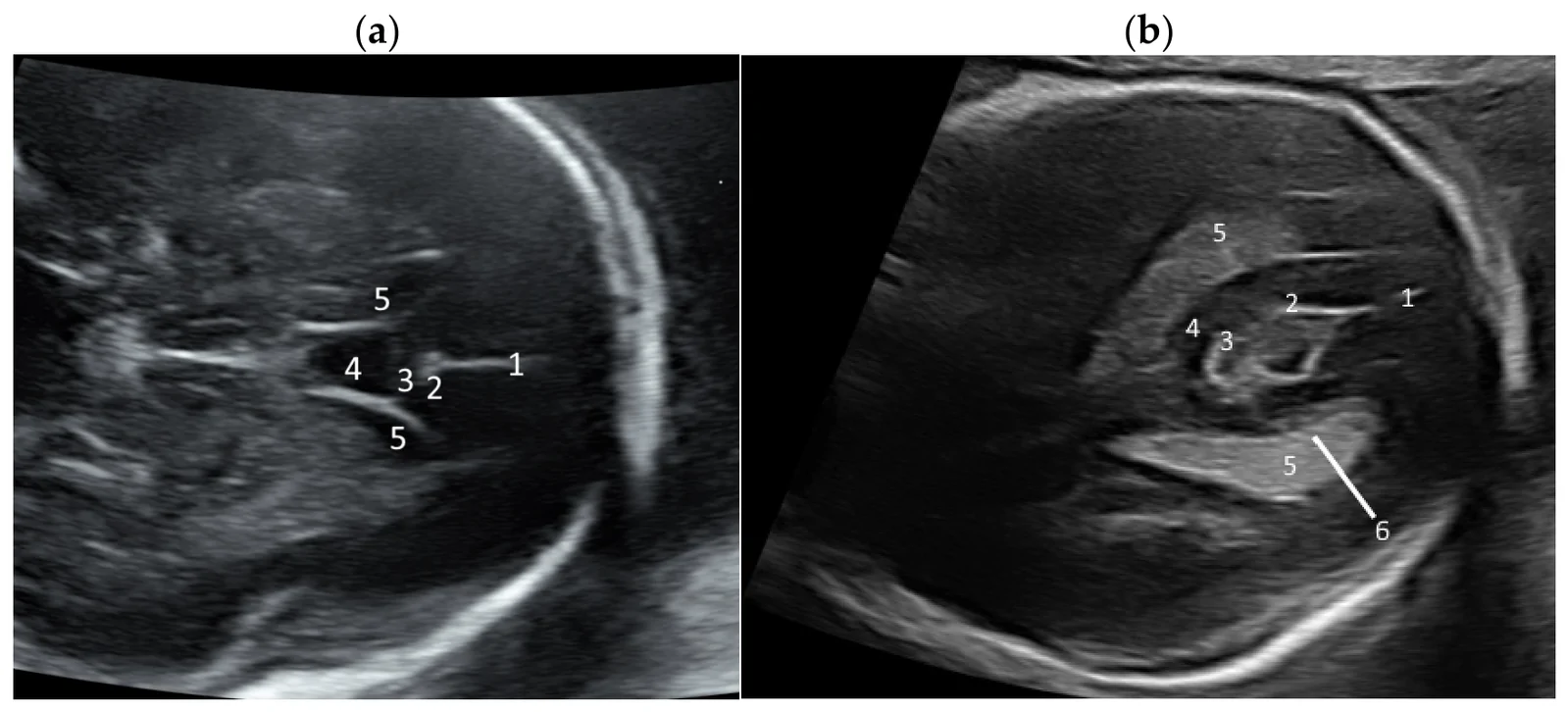
Transverse views of the foetal head, demonstrating the anterior complex (**a**) and the posterior complex (**b**) to enhance the detection of corpus callosum anomalies. Image (**a**) showing the inter-hemisphere fissure (1), the callosal sulcus (2), the genu of the corpus callosum (3), the cavum septi pellucidi (4), and the anterior horns of the lateral ventricles (5). Image (**b**) showing the inter-hemisphere fissure (1), the parieto-occipital fissure (2), the callosal sulcus (3), the splenium of the corpus callosum (4), the choroid plexus (5) and the line pointing to the medial wall of the lateral ventricle (6).

Given the limitations of the standard axial views to detect CC anomalies, evaluation of the anterior complex (AC) and the posterior complex (PC) during routine basic ultrasound examination of the foetal brain with axial views has been proposed to improve the prenatal detection of CC anomalies and other midline anomalies [17,33]. In the standard trans-ventricular plane, the anterior complex can be visualised, comprising the group of structures including, from anterior to posterior, the inter-hemisphere fissure (IHF), the pericallosal sulcus, the genu of the CC, the CSP and, laterally, the anterior horns of the lateral ventricles [17] (Figure 2a). Above the trans-ventricular plane, the posterior complex can be visualised, comprising, from anterior to posterior, the splenium of the CC, the pericallosal sulcus, the IHF, the parieto-occipital fissure and, laterally, the medial wall of the lateral ventricles [17] (Figure 2b).

In addition to the ‘indirect’ signs that can be observed in the standard axial views [24,26,27], abnormalities of the pericallosal sulcus, the genu and the splenium of the CC, the anterior horns and the medial wall of the lateral ventricle (a comma or triangular shape) can be assessed in more details in these axial views [17].

### 3.2. A Step-by-Step Approach

A step-by-step approach has been proposed to provide a detailed anatomical description of the supratentorial foetal brain, to get most information, and to assist in detection of the CNS malformations including CC anomalies using the axial views of the foetal brain during the mid-trimester screening assessment of the foetal brain [18]. This approach is based on a series of nine clinical questions, of which five are related to the detection of CC anomalies [18]. In particular, the IHF, the CSP (with length-to-width ratio ≥ 1.5), the anterior and posterior horns of the lateral ventricles, and the third ventricle are assessed in detail [18].

The presence of an echogenic mass in the IHF or a hyperechogenic CSP is a feature of lipoma of the CC [18]. Absence of the CSP and a square-shaped CSP is a feature of complete ACC and partial ACC, respectively [18]. Displacement of the anterior horns from the midline or upward displacement of the third ventricle is a feature of ACC [18].

Although a more detailed examination including evaluation of the anterior complex and the posterior complex by an expert can improve the prenatal diagnosis of CC anomalies, there are still limitations and are uncertainties whether a non-expert can identify abnormal findings as an expert [17]. More time, effort and skills are required to perform such detailed examinations. In my opinion, there are still limitations of using such detailed examinations routinely to detect CC anomalies. Whether such detailed examinations are more effective than direct visualisation of the CC to detect CC anomalies is not known. Further studies are required.

## 4. Direct Visualisation of the CC as Part of Routine Screening

### 4.1. Mid-Sagittal View

According to a recent consensus statement by a panel of international experts, 72.4% of them agreed that a sagittal view of the brain should be obtained as part of a routine screening to ensure comprehensive evaluation [25]. This was also supported by national societies [34]. Such routine direct visualisation of the CC has the potential to improve the detection rate of callosal dysgenesis [35]. However, the United Kingdom 20-week screening scan standard protocol [36], American Institute of Ultrasound in Medicine (AIUM) standard protocol [37], Australian Society of Ultrasound in Medicine (ASUM) guidelines [38], ISUOG guidelines [14], and Asia and Oceania Federation of Obstetrics and Gynaecology (AOFOG) guidelines [39] do not recommend such routine direct visualisation in low-risk populations.

In general, targeted neurosonographic examination of the CC, among other brain structures, should be performed in high-risk populations with scan findings with suspicion of a CNS anomaly, a family history of inheritable CNS malformations, a previous pregnancy affected by a foetal CNS anomaly, a present pregnancy exposure to teratogens known to affect neurogenesis, and in the presence of a foetal heart defect, infection or genetic disorder [15]. Furthermore, routine CC visualisation is performed by many professionals who practice prenatal ultrasound [32].

**Figure 3:**
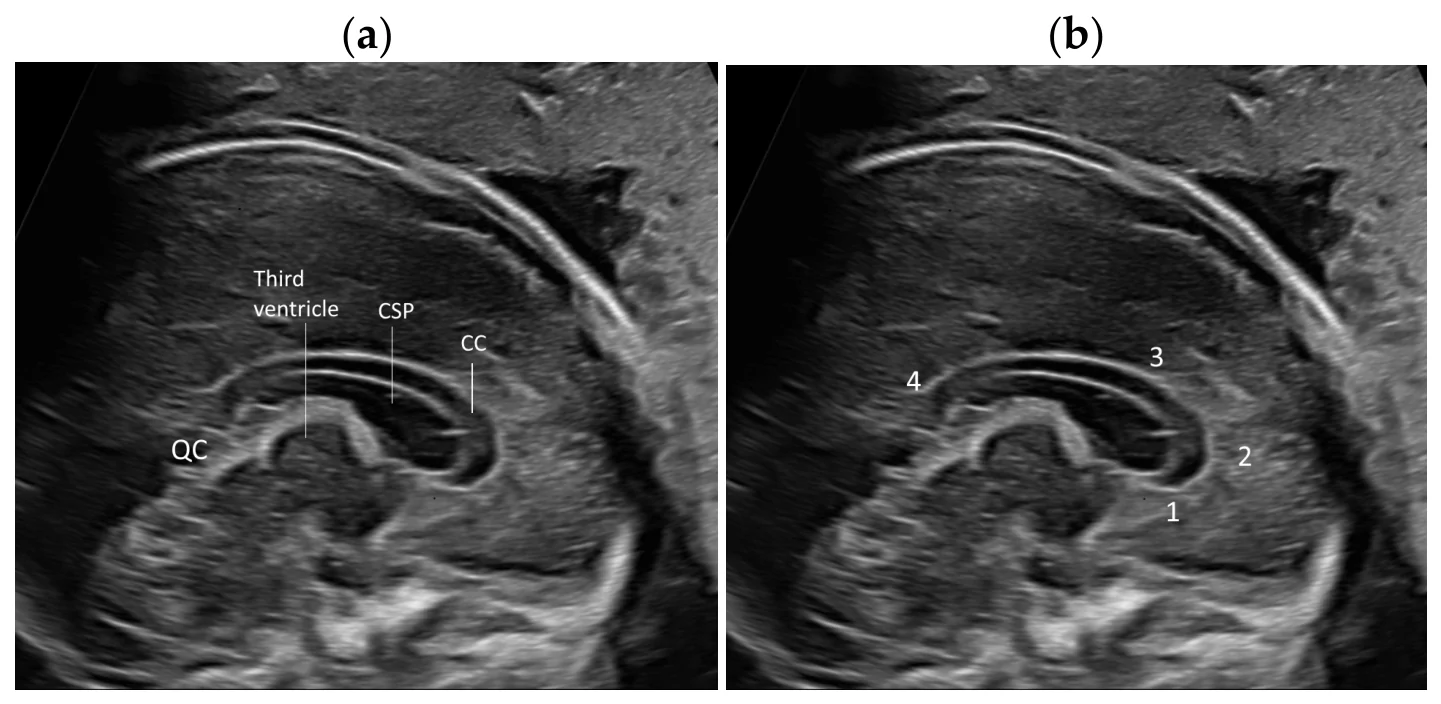
The mid-sagittal views of the foetal head (**a**,**b**), demonstrating the corpus callosum (CC), and its four components including the rostrum (1), the genu (2), the body (3) and the splenium (4) with the posterior component reaching the quadrigeminal cistern (QC). CSP, cavum septi pellucidi.

The gold standard to visualise the CC by foetal ultrasound is the mid-sagittal plane, which can allow visualisation of its entire length with four components including the rostrum, the genu, the body, and the splenium [35] (Figure 3a,b). On the other hand, only a small portion of it can be visualised in the coronal planes at a time. Coronal sonograms can show the genu of the CC that crosses the midline above the CSP and the two, closely apposed, frontal horns [5].

The mid-sagittal view of the CC can be obtained using either the transvaginal or the transfundal approach. Whatever the approach, proper alignment of an ultrasound probe along the correct scanning planes usually requires gentle manipulation of the foetal head by the free hand [32]. In foetuses in a breech presentation, a transfundal approach is used with an ultrasound probe positioning on the uterine fundus that is parallel instead of perpendicular to the abdomen [32].

### 4.2. The Quality of the CC Visualisation

The quality of the CC visualisation is affected by many factors including the foetal lie and head position, maternal body mass index (BMI), and gestational age [35]. As the foetal position is often variable throughout an ultrasound examination, an opportunity to examine the CC should be taken when the foetal position is favourable.

There are three approaches. The first is the anterior–posterior (AP) approach, which is commonly used [40]. After the foetal profile is examined in the standard sagittal view, the transducer is then angled towards the anterior fontanelle, which is used as an acoustic window to demonstrate the CC [19,35]. Fine side-to-side movements may be required to achieve an ideal image of the CC [19]. The complete visualisation rate was only 68% by the AP approach because the splenium is particularly difficult to be included in this approach [38].

The second is the cranio-caudal approach, which utilizes the craniocaudal sagittal non-ossified suture window [40]. Although such an approach is less commonly used than the AP approach, such an approach is the most successful in obtaining complete visualisation of the CC with a success rate of 90% [35,40].

The third is the posterior–anterior (PA) approach whereby the posterior fontanelle is used as an acoustic window to visualise the CC [40]. The complete visualisation rate was only 67% with the PA approach because of shadowing from the occipital bone unless the CC was imaged directly through a posterior suture [35,40].

Alternatively, a coronal section of the anterior horns of the lateral ventricles and the CSP is first obtained through the anterior fontanelle with the CSP oriented as close to vertically as possible, and the anterior horns on the same horizontal level. The transducer is then rotated 90° to obtain the mid-sagittal plane of the CC [19].

Image quality of the foetal CC can be adversely affected in obese pregnant women with a BMI ≥ 30.0 kg/m^2^ due to ultrasound beam attenuation caused by the adipose tissue of a thick abdominal wall [40]. The image quality can be optimised by (a) using a low-frequency transducer (1.5–2 MHz) to increase penetration, (b) using the transvaginal approach when the foetus is in the cephalic position, (c) alternating the woman’s position or (d) scanning through the maternal umbilicus, suprapubic area or franks to reduce the distance from the transducer to the area of interest [41]. Such skills and techniques can be developed with appropriate training [40].

As the CC develops between 12 and 16 weeks of gestation, with all components formed by 18 weeks [42], the optimal period for ultrasound examination of the foetal CC is after 20 weeks of gestation [40]. The odds of non-visualisation of the CC were 2.6 times higher before 20 weeks of gestation (7.4%) than at or after 20 weeks of gestation (3.0%) [40].

In a previous study, the rate of complete visualisation of the CC could be increased from 23% to 71% after intensive training including a didactic lecture on neuroanatomy and pathology and a one-on-one clinical training session on probe manipulation [38,43]. In another study, the rate of complete visualisation significantly improved from 71.3% to 92.1% after initial protocol implementation and training [40]. In another study, direct visualisation of the CC in a sagittal plane was achieved in 95% of cases from a cohort of 38,922 examinations [29]. The high rate of visualisation may be attributed to improved scan technique over time, as well as improving ultrasound technology with a better resolution [40].

### 4.3. The CC Biometry and Doppler

Care should be taken in using the CC biometry (length or thickness) to diagnose callosal anomalies, since a short, thin or thick CC does not necessarily mean anomalies [32]. Measurements of the CC are not mandatory for routine screening [25] because perfectly designed standards of the CC biometry have not been produced, and threshold values consistent with the rarity of anomalies have not been implemented [23]. Thus, a qualitative assessment (whether all its four components are present and normal) is much more important than a quantitative one (the biometry) [32]. Such an assessment is in line with the postnatal assessment that the CC is considered to be normally developed if its four components are present [23]. On the other hand, when there is an objective or subjective suspicion of an anomaly in the shape, length or thickness of the CC, referral to a specialist is recommended to ensure proper diagnosis and management [25].

There are two potential markers of partial ACC. The first marker is the ratio between the internal cranial occipitofrontal dimension (ICOFD) and the CC length in the mid-sagittal plane [32]. A previous study showed that such a ratio was constant throughout the pregnancy in normal populations (2.35 ± 0.11) but significantly higher in pregnancies with CC anomalies (3.20 ± 0.84) [32]. Measuring this ratio may enable rapid evaluation of the CC without the need to refer to biometry tables [32].

Another potential marker is the distance between the distal part of the CC and the choroid plexus of the third ventricle, which was relatively fixed in normal foetuses but was reduced in foetuses with partial ACC [31]. Further studies are required to evaluate the usefulness of these two potential markers.

It is feasible to use power Doppler or microvascular flow imaging to demonstrate the pericallosal artery and its branches. However, its role is marginal in the assessment of the CC [32].

### 4.4. Disadvantages of Routine Examination of the CC

There are several disadvantages of routine examination of the CC using the mid-sagittal view. First, although routine direct visualisation is feasible, obtaining the mid-sagittal view of the CC requires technical skill, and can be difficult [19]. The non-visualisation rate of the splenium with or without other components of the CC varied from 7.9% to 29% even after intensive training [35]. Second, the mean time to perform the CC views was near one minute but was not reduced even after intensive training [35]. Third, implementation of routine visualisation of the CC is difficult especially when resources or operator expertise are limited [25]. Fourth, misinterpretation may lead to overdiagnosis and unnecessary parental anxiety. Thus, interpretation of the mid-sagittal view should be cautious, taking into account the full clinical context, local infrastructure and counselling capacities [25].

It is difficult to visualise the CC completely, quickly and easily in a screening examination because of an unfavourable foetal position [35]. As time is limited in a screening examination, it is not justified to take a long time to get difficult views of the CC at the expense of the examination of other important structures. In my opinion, alternatively, a more detailed examination of the foetal brain by axial planes including assessment of the anterior and posterior complex, and the step-by-step approach as discussed above can be performed carefully to look for indirect signs of callosal anomalies [17,18,33]. Such an alternative approach can avoid recalling the patient for assessment of the CC alone and the related cost. If the routine sagittal CC view is to be implemented, training, audits and quality management of this examination are required [35].

In view of the above disadvantages, implementation of routine direct examination of the CC remains a challenge to the clinicians or sonographers. Unlike the targeted neurosonography performed by experts using high-end ultrasound machines with sufficient scanning time, the routine mid-trimester screening examination for low-risk pregnant women is usually performed by sonographers who are often not an expert, are allocated only a very limited time, and are without a high-end ultrasound machine [35,43]. Furthermore, if such a routine direct examination is implemented, there will be medico-legal implications when CC anomalies are missed prenatally, and resources implications when a rescan is performed for non-visualisation of the CC [35]. In my opinion, routine direct examination of the CC in low-risk populations should be flexible according to the local circumstances rather than mandatory unless an easy and accurate examination method is available.

### 4.5. Transvaginal Approach

Although the transvaginal approach is the preferred method to perform an adequate high-resolution targeted neurosonographic examination [44], the systematic use of such an approach is not necessary in the standard screening practice [25]. When a foetus is in the vertex presentation, and the transabdominal views are inadequate or nondiagnostic, the transvaginal approach provides significant advantages over the transabdominal one including a higher resolution, due to a higher emission frequency, and scanning through sagittal and coronal planes to avoid acoustic shadowing produced by the calvarium [32]. In a previous study, the transvaginal approach was required in a small proportion (4.4%) of the second trimester screening examinations [40].

## 5. Use of 3D Reconstruction

**Figure 4:**
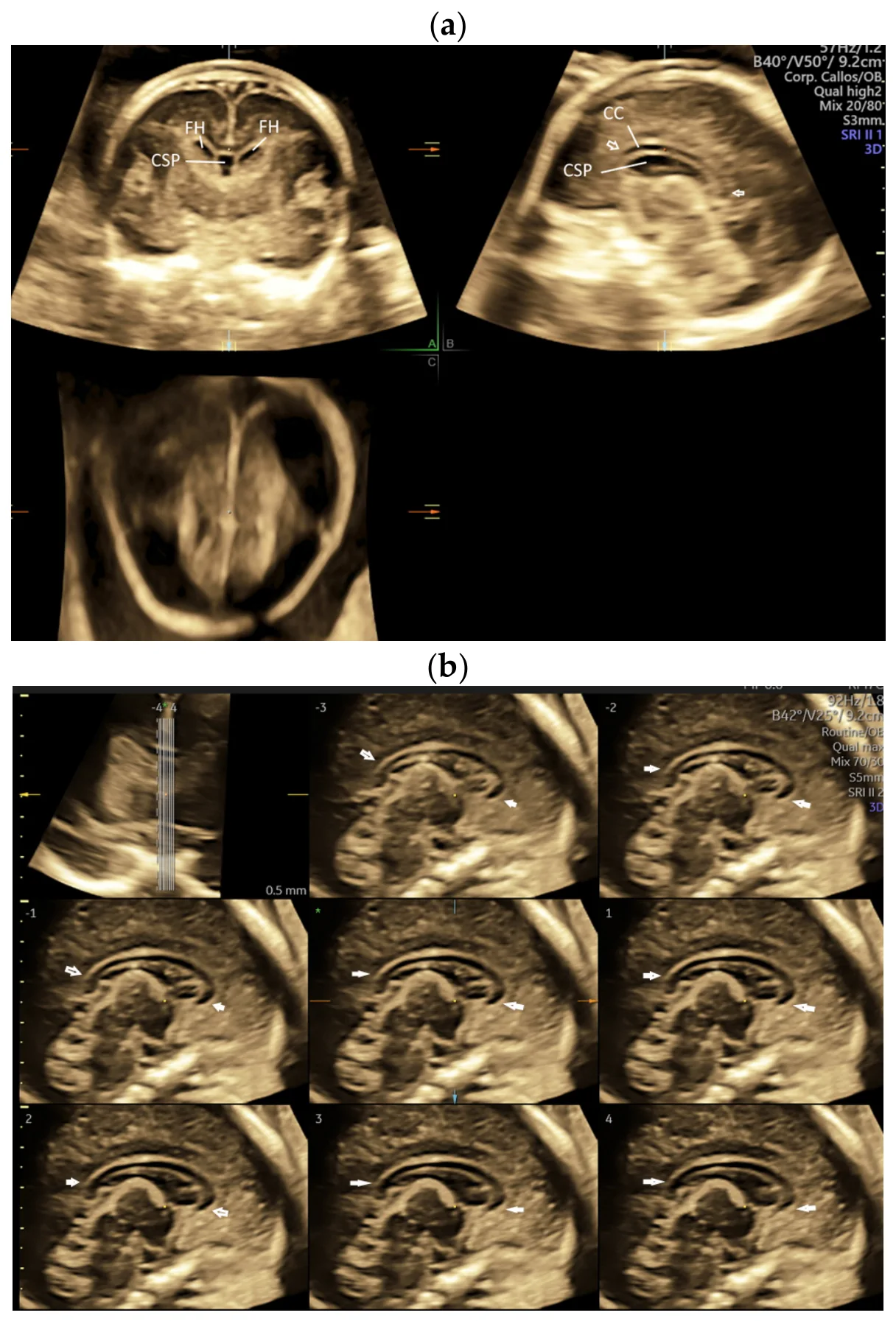
Three-dimensional multiplanar image of the foetal brain (**a**) with a volume acquisition at plane A, and its two reconstructed (arrows) orthogonal planes (B and C). Coronal plane (Plane (A)) shows part of the corpus callosum (dot), the frontal horns (FHs) of the lateral ventricles, on either side of the cavum septi pellucidi (CSP). The mid-sagittal plane (Plane (B)) shows the corpus callosum (CC) (arrows). Three-dimensional tomographic multi-slice imaging of the mid-sagittal plane of the foetal brain with Volume Contrast Imaging activated (**b**) showing the CC (arrows). The level of the multiple slices are indicated in the left upper corner image from ‘−3’ … ‘*’ … to ‘4’.

Although systematic use of a 3D ultrasound approach is not necessary in the standard screening practice [25], 3D ultrasound reconstruction may be useful in selected cases when difficulties in obtaining a sagittal view of the CC through 2D ultrasound are encountered. 3D ultrasound allows the acquisition of volumes in the standard axial planes with subsequent multiplanar reconstruction of the mid-sagittal plane [44] (Figure 4a). Furthermore, the quality of 3D images of the CC can be improved by multiplanar reconstruction techniques like VCI (Volume Contrast Imaging) mode, which allows displaying thicker ‘slices’ of the CC and enhancing the signal-to-background noise ratio [32] (Figure 4b).

The extent to which the CC can be assessed in 3D reconstructed sagittal planes is entirely dependent on the plane of a volume acquisition [45]. The 3D mid-sagittal reconstructed plane will likely be obtained if the 3D volume is acquired at the mid-sagittal plane with the angle between the transducer and the direction of the foetal nose ranging from 0° to 179° and from 330° to 359° [45]. Another study also showed that the best technique for visualisation of the CC in a single image involved 3D acquisition in a sagittal plane through the sagittal suture [44]. These are the planes at which the CC can be visualised by 2D ultrasound though the metopic suture, anterior fontanelle, sagittal suture or posterior fontanelle as discussed in the Section 4. In my opinion, these results are expected as obtaining a good 2D view before volume acquisition is a pre-requisite to obtaining good 3D images after reconstruction.

The 3D mid-sagittal reconstructed plane will also likely be obtained if the 3D volume is acquired at an oblique plane around the crown–rump axis with an angle from the mid-sagittal plane of less than 30° [45]. Thus, in my opinion, when the 2D plane is still slightly oblique around the crown–rump axis with an angle from but not exactly at the mid-sagittal plane despite an appropriate manipulation by the free hand, a 3D volume acquisition can be attempted to get the mid-sagittal reconstructed view subsequently. This may save time and effort in getting the mid-sagittal view of the CC through 2D ultrasound.

However, if a 3D volume is acquired at the axial BPD plane [46], reconstruction of the mid-sagittal plane will likely show a hyperechogenic artifact instead of the CC, which should be anechoic or hypoechogenic [47]. Such a hyperechogenic artifact can be confused with a hyperechogenic CC, which is a feature of callosal lipoma [47]. In another study, a 3D volume acquisition from an axial plane at the level of the BPD plane with an angle of 90° to the midline is insufficient to evaluate the CC [48].

There are several disadvantages of such a 3D approach. First, some sonologists may not be familiar with the use of a 3D ultrasound and its related multiple processing techniques [49]. These techniques are not easy to learn and are time consuming in the absence of specific training [49]. Second, the quality of the 3D volume acquired depends on several factors including maternal obesity, depth of imaging, foetal lie, foetal movements, angle and speed of a volume acquisition [49]. Third, artifacts in the 3D ultrasound may result in image distortions or misrepresentations that can obscure or mimic pathology.

In my opinion, a 3D ultrasound approach should be encouraged especially when it is difficult to obtain a good 2D image of the CC or in high-risk populations. 3D ultrasound can display the true mid-sagittal plane of the brain including CC and can enhance its image quality by displaying thicker ‘slices’ of the CC [32].

## 6. New Insights and Future Research Directions

In a previous study, when the posterior–anterior approach or anterior–posterior approach was used, complete visualisation of the CC was achieved in only 67% or 68% of the studied pregnancy women [35]. Despite visualising three of the four components of the CC, the splenium was not visualised in 13% of the studied pregnant women even after intensive training [35]. Efforts should be made to image the splenium, which is commonly absent in partial ACC. Alternatively, the splenium can be visualised when the posterior complex is examined [17].

**Figure 5:**
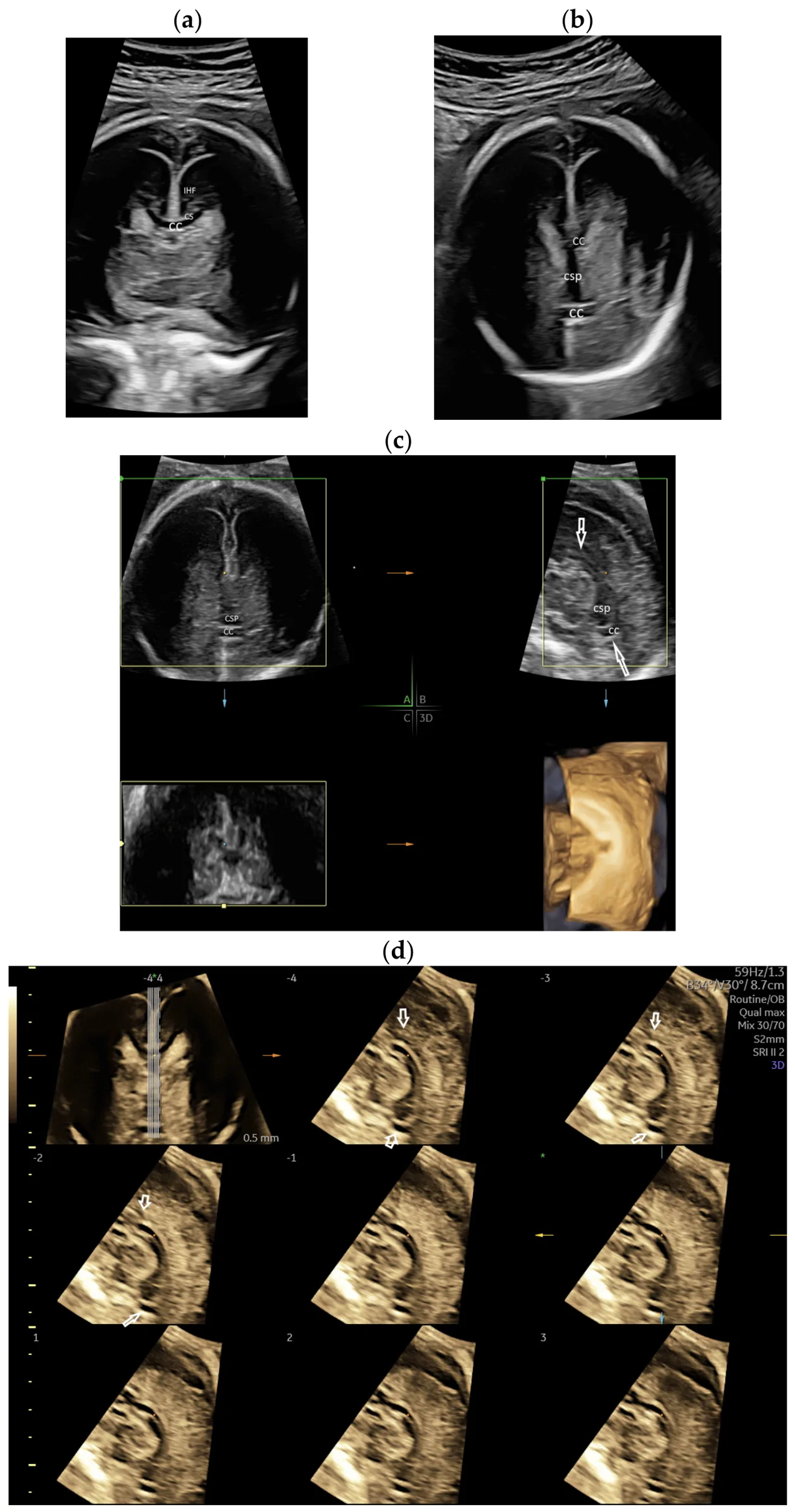
Transverse view of the foetal head with the transducer facing the posterior fontanelle showing the splenium of the corpus callosum (CC) (**a**), with both the rostrum and the splenium simultaneously (**b**). Three-dimensional multiplanar image of the foetal brain following a volume acquisition at Plane (A) showing the reconstructed (color arrow) mid-sagittal view (arrows) as an anechoic shadow in Plane (B) (**c**). Three-dimensional tomographic multi-slice imaging of the mid-sagittal plane of the foetal brain with Volume Contrast Imaging activated showing the CC (arrows) (**d**). CSP, cavum septi pellucidi. The level of the multiple slices are indicated in the left upper corner image from ‘−4’ … ‘*’ … to ‘3’.

In my experience, from an axial view at the level of the posterior complex, rocking the transducer to face the posterior fontanelle can show the splenium between the echogenic pericallosum sulcus and the anechoic cavum vergae (Figure 5a). Similarly, from an axial view at the level of the anterior complex, rocking the transducer to face the anterior fontanelle can show the rostrum between the echogenic pericallosum sulcus and the anechoic CSP. In either of these two views, fanning the transducer can show both the rostrum and the splenium simultaneously. Rotating the transducer 90° can then show the mid-sagittal plane of the CC. If a 3D volume is acquired at either of these two tilted axial planes with both the rostrum and the splenium in view, the 3D reconstructed mid-sagittal view of the CC will be visualised as an anechoic shadow (Figure 5b–d).

In a previous study, the CC visualisation rate was 42% when a 3D volume was acquired axially at the level of BPD plane with an angle of 0–29 degrees from the midline [45]. Further studies are required to investigate whether these proposed new 2D or 3D approaches (as discussed above) in combination with other approaches can improve the visualisation rate of the CC in difficult situations.

Recently, a deep learning-based artificial intelligence (AI)-assisted diagnostic framework CC-FocusNet for the automated classification of CC developmental status has been investigated [50]. As the present screening examination is based on axial views of the foetal brain, and some indirect signs of the CC anomalies are too subtle to an experienced sonographer, it is worthwhile to investigate whether a deep learning machine can detect these subtle indirect signs by analysing a video clip of a slow axial sweep of the foetal brain.

### Pragmatic Approach

Current guidelines recommend direct examination of the CC by the mid-sagittal view in high-risk populations [15,25,37]. In my opinion, in view of the current evidence, when resources or operator expertise are limited, a pragmatic approach is to obtain the mid-sagittal plane of the CC as optional (when technically feasible) in low-risk populations. When difficulties are encountered in obtaining the mid-sagittal view, selective use of (a) the transvaginal approach when the foetus is in a cephalic presentation, or (b) 3D reconstructed plane after a volume acquisition in an appropriate image plane can increase the complete visualisation rate of the CC [45].

According to the consensus statement, if the axial views of the foetal brain are normal, it is not mandatory or advisable to perform a repeat scan or prolonged scan for the mid-sagittal view of the CC in a routine screening setting [25]. In my opinion, when it is technically not feasible to obtain the mid-sagittal view of the CC within a limited time, in addition to the standard axial views of the foetal brain, it is beneficial to examine the anterior and posterior complexes, and to get the most information from the axial views by a step-by-step approach to detect indirect signs of CC anomalies, which may be subtle [17,18]. If any abnormal or suspicious sign is present, targeted neurosonography is required [32]. Whether such an approach is effective in screening for CC anomalies need further studies.

## 7. Conclusions

In this review article, various prenatal ultrasound screening methods of CC anomalies including the standard axial views, more detailed axial views, the mid-sagittal view, the transvaginal approach, and 3D reconstruction are discussed. Key features of these methods are summarised in Table 1. Comparison of various guidelines/consensus statement [14,15,25,34,36,37,38,39], and the performance of these methods [16,28,29,33,35,40,44,45,51] are summarised in Table 2 and Table 3, respectively. New insights on screening methods are also shared. Further studies are required to investigate new screening planes, deep learning machines or the pragmatic approach to improve the prenatal detection of CC anomalies over the standard axial views.

## Figures and Tables

**Table 1:** Key features of various screening methods for corpus callosum (CC) anomalies.

Direct Visualisation of CC	Approach	Key Features
No	Standard axial views of the foetal brain	Look for indirect signs including an abnormal or absent CSP, dilatation, or a tear-shaped appearance and malalignment of the lateral ventricles, a high-riding and dilated third ventricle, and a widened IHF
No	Examination of the AC and PC	In addition to the above indirect signs, abnormalities of the pericallosal sulcus, the genu and the splenium of the CC, the anterior horns and the medial wall of the lateral ventricle can also be assessed
No	A step-by-step approach based on a series of questions	Assessment of (a) the IHF, (b) the CSP, (c) the anterior horns of the lateral ventricles, (d) the third ventricle, and (e) the posterior horns of the lateral ventricles
Yes	2DUS, transfundal, mid-sagittal plane	Feasible but can be difficult in screening examination Qualitative assessment of the four components of the CC Measurement of the CC or assessment of the pericallosal artery is not mandatory
Yes	2DUS, transvaginal	As above In selected cases when the foetus is in the vertex presentation, and the transabdominal views are inadequate or nondiagnostic
Yes	3DUS, volume acquired at mid-sagittal plane	In selected cases when difficulties in obtaining a sagittal view of the CC through 2DUS is encountered The extent to which the CC can be assessed in 3D reconstructed sagittal planes is entirely dependent on the plane of a volume acquisition The quality of the 3D images of the CC can be improved by multiplanar reconstruction techniques like Volume Contrast Imaging mode or Omni View technology.

AC, anterior complex; CC, corpus callosum; CSP, cavum septi pellucidi; IHF, inter-hemisphere; PC, posterior complex; 2D, two-dimensional; 3D, three-dimensional; US, ultrasound.

**Table 2:** Comparison of international and national guidelines or consensus statements on routine prenatal ultrasound screening for corpus callosum (CC) anomalies.

Guidelines or Consensus Statement	The Routine Mid-Sagittal View of the CC, in Addition to the Standard Transverse Views
Consensus statement by a panel of international experts [25]	Yes for all
National societies [37]	Yes for all
ISUOG guidelines on targeted neurosonography [15]	Yes for high -risk populations
AIUM standard protocol on detailed examination [33]	Yes for high -risk populations
ISUOG guidelines on the routine mid-trimester foetal ultrasound examination [14]	No for low-risk populations
AIUM standard protocol on standard examination [33]	No for low-risk populations
ASUM guidelines on the routine mid-trimester scan [34]	Not specified
United Kingdom 20-week screening scan standard protocol [32]	Not specified
AOFOG guidelines on the mid-trimester routine scan [35]	Not specified

AIUM, American Institute of Ultrasound in Medicine; AOFOG, Asia and Oceania Federation of Obstetrics and Gynaecology; ASUM, Australian Society of Ultrasound in Medicine; ISUOG, International Society of Ultrasound in Obstetrics and Gynecology.

**Table 3:** Comparison of performance in detecting agenesis of corpus callosum (ACC) or visualisation of the corpus callosum (CC) among various imaging approaches.

Imaging Approaches	Performance
Standard axial views	Sensitivity and specificity of detecting ACC [16] - 72% and 98%, respectively (overall) - 84% and 98%, respectively (neurosonography) - 57% and 89%, respectively (screening) Proportion of abnormal signs in cases of partial ACC [28] - Abnormal CSP: 86.4% - Distension of IHF: 77.1% - Dilated and elevated third ventricle: 47.4% - Ventriculomegaly: 35.6%
Assessment of AC and PC	Proportion of abnormal signs in cases of ACC [36] - Abnormal AC: 5/5 - Abnormal PC (widening IHF or non-visualisation of splenium): 5/5 Proportion of abnormal signs in cases of partial ACC [51] - Abnormal CSP: 20/22 - Dysmorphic anterior horn: 11/22 - Widening of IHF in PC: 20/22 - Absent callosal sulcus without CC cross midline in PC: 19/22
Step by step approach	No data
Mid-sagittal view	Visualisation rate of CC [29,38,40]: - 23% to 71.3% (before training) - 71% to 95% (after training) Non-visualisation rate of splenium with or without other components of the CC: 7.9% to 29%. No data of sensitivity or specificity of detecting ACC
3D ultrasound reconstruction	Visualisation rate of CC using 3D reconstruction after a volume acquisition in the mid-sagittal view [44,45]: 83% to 93%.

AC, anterior complex; CSP, cavum septi pellucidi; IHF, inter-hemisphere fissure; PC, posterior complex; 3D, Three-dimensional.

## Data Availability

No new data were created or analyzed in this study. Data sharing is not applicable to this article.

## Abbreviations

The following abbreviations are used in this manuscript:

AP	Anterior–posterior
CC	Corpus callosum
CSP	Cavum septi pellucidi
IHF	Inter-hemisphere fissure
PA	Posterior-anterior
2D	Two-dimensional
3D	Three-dimensional

## References

[1] Sperry R.W. (1968). Hemispheric disconnection and unity in conscious awareness. Am. Psychol..

[2] Raybaud C. (2010). The corpus callosum, the other great forebrain commissures, and the septum pellucidum: Anatomy, development and malformation. Neuroradiology.

[3] Santo S., D’Antonio F., Homfray T., Rich P., Pilu G., Bhide A., Thilaganathan B., Papageorghiou A.T. (2012). Counseling in fetal medicine: Agenesis of the corpus callosum. Ultrasound Obstet. Gynecol..

[4] Loeser J.D., Alvord E.C. (1968). Agenesis of the corpus callosum. Brain.

[5] Atlas S.W., Shkolnik A., Naidich T.P. (1985). Sonographic Recognition of Agenesis of the Corpus Callosum. AJNR Am. J. Neuroradiol..

[6] Chiappedi M., Fresca A., Baschenis I.M. (2012). Complete corpus callosum agenesis: Can it be mild?. Case Rep. Pediatr..

[7] Sotiriadis A., Makrydimas G. (2012). Neurodevelopment after prenatal diagnosis of isolated agenesis of the corpus callosum: An integrative review. Am. J. Obstet. Gynecol..

[8] De Keersmaecker B., Jansen K., Aertsen M., Naulaers G., De Catte L. (2024). Outcome of partial agenesis of corpus callosum. Am. J. Obstet. Gynecol..

[9] Palmer E.E., Mowat D. (2014). Agenesis of the corpus callosum: A clinical approach to diagnosis. Am. J. Med. Genet. C Semin. Med. Genet..

[10] Mustafa H.J., Barbera J.P., Sambatur E.V., Pagani G., Yaron Y., Baptiste C.D., Wapner R.J., Brewer C.J., Khalil A. (2024). Diagnostic yield of exome sequencing in prenatal agenesis of corpus callosum: Systematic review and meta-analysis. Ultrasound Obstet. Gynecol..

[11] Bernardes Da Cunha S., Carneiro M.C., Miguel Sa M., Rodrigues A., Pina C. (2021). Neurodevelopmental outcomes following prenatal diagnosis of isolated corpus callosum agenesis: A systematic review. Fetal Diagn. Ther..

[12] Tsai P., Shinar S. (2023). Agenesis of the corpus callosum: What to tell expecting parents?. Prenat. Diagn..

[13] Huang R., Chen J., Hou X., Liu L., Sun G., Pan H., Ma Y. (2024). Retrospective analysis of the prognostic factors of fetal corpus callosum dysplasia. BMC Pregnancy Childbirth.

[14] Salomon L.J., Alfirevic Z., Berghella V., Bilardo C.M., Chalouhi G.E., Da Silva Costa F., Hernandez-Andrade E., Malinger G., Munoz H., Paladini D. (2022). ISUOG Practice Guidelines (updated): Performance of the routine mid-trimester fetal ultrasound scan. Ultrasound Obstet. Gynecol..

[15] Malinger G., Paladini D., Haratz K.K., Monteagudo A., Pilu G.L., Timor-Tritsch I.E. (2020). ISUOG Practice Guidelines (updated): Sonographic examination of the fetal central nervous system. Part 1: Performance of screening examination and indications for targeted neurosonography. Ultrasound Obstet. Gynecol..

[16] Zhang Y. (2023). Prenatal ultrasound for the diagnosis of the agenesis of corpus callosum: A meta-analysis. J. Matern. Fetal Neonatal Med..

[17] Vinals F., Correa F., Gonçalves-Pereira P.M. (2015). Anterior and posterior complexes: A step towards improving neurosonographic screening of midline and cortical anomalies. Ultrasound Obstet. Gynecol..

[18] Della Valle L., Pilu G., Khalil A., Rizzo G., Pooh R., Galindo A., Grisolia G., Duncan J.R., Timor-Tritsch I.E., D’Antonio F. (2026). How to get the most from axial views of the fetal brain: Applying the principles of neurosonography to screening examination of the supratentorial central nervous system. Ultrasound Obstet. Gynecol..

[19] Youssef A., Ghi T., Pilu G. (2013). How to image the fetal corpus callosum. Ultrasound Obstet. Gynecol..

[20] Miguelote R.F., Vides B., Santos R.F., Palha J.A., Matias A., Sousa N. (2011). The role of three-dimensional imaging reconstruction to measure the corpus callosum: Comparison with direct mid-sagittal views. Prenat. Diagn..

[21] Rizzo G., Abuhamad A.Z., Benacerraf B.R., Chaoui R., Corral E., Addario V.D., Espinoza J., Lee W., Merce Alberto L.T., Pooh R. (2011). Collaborative study on 3-dimensional sonography for the prenatal diagnosis of central nervous system defects. J. Ultrasound Med..

[22] Mahallati H., Sotiriadis A., Celestin C., Millischer A.E., Sonigo P., Grevent D., O’Gorman N., Bahi-Buisson N., Attie-Bitach T., Ville Y. (2021). Heterogeneity in defining fetal corpus callosal pathology: Systematic review. Ultrasound Obstet. Gynecol..

[23] Salomon L.J., Paladini D. (2024). Fetal corpus callosal anomalies: From disease of classification to classification of disease. Ultrasound Obstet. Gynecol..

[24] Paladini D., Pastore G., Cavallaro A., Massaro M., Nappi C. (2013). Agenesis of the fetal corpus callosum: Sonographic signs change with advancing gestational age. Ultrasound Obstet. Gynecol..

[25] Corroenne R., Paladini D., Papapastefaniou I., Chaoui R., Pomar L., Guibaud L., Krajden Haratz K., Pooh R.K., Herrera M., Xemines R. (2025). Prenatal evaluation, diagnosis and management of fetal corpus callosal abnormalities: International Delphi consensus. Obstet. Gynecol..

[26] Shen O., Gelot A.B., Moutard M.L., Jouannic J.M., Sela H.Y., Garel C. (2015). Abnormal shape of the cavum septi pellucidi: An indirect sign of partial agenesis of the corpus callosum. Ultrasound Obstet. Gynecol..

[27] Pilu G., Sandri F., Perolo A., Pittalis M.C., Grisolia G., Cocchi G., Foschini M.P., Salvioli G.P., Bovicelli L. (1993). Sonography of fetal agenesis of the corpus callosum: A survey of 35 cases. Ultrasound Obstet. Gynecol..

[28] Zhou C., Li H., Han R., Ren H., Shen B., Wang X., Feng F., Wang M., Liu L. (2025). Partial agenesis of the corpus callosum: Prenatal ultrasound characteristics, associations, and outcome. Acta Obstet. Gynecol. Scand..

[29] Rodriguez M., Echevarria M., Perdomo L., Gómez-Chiari M., García S., Prats P., Serra B., Albaiges G. (2024). Prevalence of corpus callosum pathology in an unselected population. Should assessment of the corpus callosum be included in the routine 20 weeks scan?. Prenat. Diagn..

[30] Ghi T., Carletti A., Contro E., Cera E., Falco P., Tagliavini G., Michelacci L., Tani G., Youssef A., Bonasoni P. (2010). Prenatal diagnosis and outcome of partial agenesis and hypoplasia of the corpus callosum. Ultrasound Obstet. Gynecol..

[31] Stanislao V., Rizzo G., Grisolia G., Raffaelli R., Arduini M., Pinto A., Mappa I., Pisello M., Patelli C., Mazzucca B. (2025). Corpus Callosum to Choroid Plexus Distance for the Prenatal Diagnosis of Partial Agenesis or Hypoplasia of the Corpus Callosum: A Multicenter Study. J. Ultrasound Med..

[32] Paladini D., Malinger G., Birnbaum R., Monteagudo A., Pilu G., Salomon L.J., Timor-Tritsch I.E. (2021). ISUOG Practice Guidelines (updated): Sonographic examination of the fetal central nervous system. Part 2: Performance of targeted neurosonography. Ultrasound Obstet. Gynecol..

[33] Cagneaux M., Guibaud L. (2013). From cavum septi pellucidi to anterior complex: How to improve detection of midline cerebral anomalies. Ultrasound Obstet. Gynecol..

[34] Conference Nationale d’Echographie Obstétricale et Foetale. https://www.ordre-sages-femmes.fr/wp-content/uploads/2022/10/CNEOF-19-octobre-2022_compressed-1-2.pdf.

[35] Lavender I., Coombs P.R., Haltren K.V., Robinson A.J. (2016). Routine screening for callosal dysgenesis in the second trimester is achievable with intensive training. J. Ultrasound Med..

[36] National Health Service (NHS) 20-Week Screening Scan. Updated 12 October 2022. Reviewed 2024. https://www.nhs.uk/pregnancy/your-pregnancy-care/20-week-scan/.

[37] American Institute of Ultrasound in Medicine (AIUM) (2019). AIUM Practice Parameter for the Performance of Detailed Second- and Third-Trimester Diagnostic Obstetric Ultrasound Examinations. J. Ultrasound Med..

[38] Australasian Society for Ultrasound in Medicine (ASUM) Guidelines for the Performance of Second (Mid) Trimester Ultrasound. Revised 2018. https://www.asum.com.au/standards-of-practice/.

[39] Leung K.Y., Poon C.F., Teotico A.R., Hata T., Won H.S., Chen M., Chittacharoen A., Malhotra J., Shah P.K., Salim A. (2015). Recommendations on routine mid-trimester anomaly scan. J. Obstet. Gynaecol. Res..

[40] Fernando S., Lavender I., Coombs P., Van Haltren K., Lombardo P., Goodyear M., Rolnik D.L.J. (2025). Visualization of the Fetal Corpus Callosum in Routine Second Trimester Screening Ultrasound Examinations. Ultrasound Med..

[41] Aberle D., Charles H., Hodak S., O’Neill D., Oklu R., Deipolyi A. (2017). Optimizing care for the obese patient in interventional radiology. Diagn. Interv. Radiol..

[42] Malinger G., Zakut H. (1993). The corpus callosum: Normal fetal development as shown by transvaginal sonography. Am. J. Roentgenol..

[43] Nicholls D., Sweet L., Hyett J. (2014). Psychomotor skills in medical ultrasound imaging: An analysis of the core skill set. J. Ultrasound Med..

[44] Frisovaa V., Srutovac M., Hyett J. (2018). 3-D Volume Assessment of the Corpus Callosum and Cerebellar Vermis Using Various Volume Acquisition and Post-Processing Protocols. Fetal Diagn. Ther..

[45] Plasencia W., Dagklis T., Borenstein M., Csapo B., Nicolaides K.H. (2007). Assessment of the corpus callosum at 20–24 weeks’ gestation by three-dimensional ultrasound examination. Ultrasound Obstet. Gynecol..

[46] Muscatello A., Accurti V., Conte V., Franchi V., Colagrande I., Carta A., Patacchiola F., Palermo P., Carta G. (2013). Comparison between Two- and Three-Dimensional Ultrasound in Visualization of Corpus Callosum during Second Trimester Routine Scan: Our Experience. Fetal Diagn. Ther..

[47] Malinger G., Lerman-Sagie T., Viñals F. (2006). Three-dimensional sagittal reconstruction of the corpus callosum: Fact or artifact?. Ultrasound Obstet. Gynecol..

[48] Pashaj S., Merz E., Wellek S. (2013). Biometry of the fetal corpus callosum by three-dimensional ultrasound. Ultrasound. Obstet. Gynecol..

[49] Dall’Asta A., Paramasivam G., Basheer S.N., Whitby E., Tahir Z., Lees C. (2019). How to obtain diagnostic planes of the fetal central nervous system using three-dimensional ultrasound and a context-preserving rendering technology. Am. J. Obstet. Gynecol..

[50] Li M., Liu S., Zhang Z., Li Q., Xu X. (2026). Deep learning-based automated detection of fetal corpus callosum abnormalities in prenatal ultrasound. Front. Pediatr..

[51] Vinals F., Correa F., García-Rodríguez R., Tubau-Navarra A., Esparza M., Diaz L. (2025). Anomalies on anterior and posterior complex views in fetuses with partial agenesis of corpus callosum. Ultrasound Obstet. Gynecol..

